# Estimating health workforce needs for antiretroviral therapy in resource-limited settings

**DOI:** 10.1186/1478-4491-4-1

**Published:** 2006-01-26

**Authors:** Lisa R Hirschhorn, Lulu Oguda, Andrew Fullem, Norbert Dreesch, Paul Wilson

**Affiliations:** 1 JSI Research and Training Institute, Inc., Boston, Massachusetts, USA; 2 Harvard Medical School Division of AIDS, Boston, Massachusetts, USA; 3 BroadReach Healthcare, LLC, Washington, DC, USA; 4 World Health Organization, Department of Human Resources for Health, Geneva, Switzerland; 5 Center for Global Health and Economic Development, Columbia University, New York, New York, USA

## Abstract

**Background:**

Efforts to increase access to life-saving treatment, including antiretroviral therapy (ART), for people living with HIV/AIDS in resource-limited settings has been the growing focus of international efforts. One of the greatest challenges to scaling up will be the limited supply of adequately trained human resources for health, including doctors, nurses, pharmacists and other skilled providers. As national treatment programmes are planned, better estimates of human resource needs and improved approaches to assessing the impact of different staffing models are critically needed. However there have been few systematic assessments of staffing patterns in existing programmes or of the estimates being used in planning larger programmes.

**Methods:**

We reviewed the published literature and selected plans and scaling-up proposals, interviewed experts and collected data on staffing patterns at existing treatment sites through a structured survey and site visits.

**Results:**

We found a wide range of staffing patterns and patient-provider ratios in existing and planned treatment programmes. Many factors influenced health workforce needs, including task assignments, delivery models, other staff responsibilities and programme size. Overall, the number of health care workers required to provide ART to 1000 patients included 1–2 physicians, 2–7 nurses, <1 to 3 pharmacy staff, and a much wider range of counsellors and treatment supporters. We estimate from these data that the equivalent of 20 000 to 100 000 physicians, nurses, pharmacists and other core clinical staff will be needed to meet the WHO target of treating 3 million people by the end of 2005. The total number of staff, including counsellors, administrators and other cadres, could be substantially higher.

**Discussion:**

These data are consistent with other estimates of human resource requirements for antiretroviral therapy, but highlight the considerable variability of current staffing models and the importance of a broad range of factors in determining personnel needs. Few outcome or cost data are currently available to assess the effectiveness and efficiency of different staffing models, and it will be important to develop improved methods for gathering this information as treatment programmes are scaled up.

## Background

The availability of effective treatment for HIV/AIDS through antiretroviral therapy (ART) has resulted in dramatic declines in morbidity, mortality and mother-to-child transmission (MTCT) in resource-rich countries [1,2]. Similar results have been reported in well-designed and -staffed programmes providing ART in resource-limited settings [3,4]. Decreased cost of antiretrovirals (ARVs) combined with simpler recommendations for initiation and monitoring from the World Health Organization (WHO), has led to international efforts to make ART available to all those in clinical need. However, as ARV costs fall, the shortage of human resources for health (HRH), including trained medical and support staff, will continue to be a formidable obstacle to providing effective ART in resource-limited settings.

The regions in greatest need of comprehensive treatment programmes overlap with those that already face significant HRH deficits, and most ART scale-up efforts will face competing demands for trained staff both locally and regionally. Causes of these deficits have been well described and include the following:

• low salaries and poor working conditions leading to exodus from the public sector health care system;

• unequal geographical distribution of health staff within a country or district (rural versus urban);

• morbidity and mortality of health care workers living with HIV/AIDS;

• absence from work in order to care for sick family members or attend funerals;

• HIV-related stigma resulting in reluctance to care for people living with HIV/AIDS (PLWHAs);

• regulations restricting critical activities (e.g. dispensing, prescribing, laboratory analyses) to specific cadres of health personnel;

• long-standing fiscal constraints impeding the rapid expansion of the public health workforce and the adjustment of remuneration packages towards competitive levels;

• inadequate in-country capacity to train new health care workers [5].

While some systematic assessments have been published on how HRH deficits may affect HIV care including ART, work is continuing at the local, country and international levels to more completely assess current capacity and future HRH requirements [5-7]. WHO estimates that 100 000 health care workers will need to be trained to meet the "3 by 5" goals of treating 3 million with ART by 2005 [8]. Understanding the gap between current HRH availability and current and future needs and developing strategies to address these gaps are crucial to planning for expanded access to treatment, and these issues have been the focus of a number of reports [6,9-13].

In this paper, we review organizational factors that influence HRH requirements and use data from existing and planned treatment programmes to develop estimates of human resources needs for a scale-up of ART in resource-limited settings.

## Methods

### Estimations of HRH requirements

Several methods exist for estimating HRH needs for delivering HIV care including ART. These approaches (or elements of an approach) include:

• population ratios: based on ratios of providers to the entire population;

• health needs: based on known health needs of a population;

• demands for services or targets for services: based on quantitative goals for delivery of specific services (e.g. per cent vaccinated), which are then translated into HRH requirements based on staffing and estimates of productivity;

• job-based: based on estimating the number of staff belonging to specific cadres (physicians, nurses, etc.) required to serve a given number of patients;

• task- or function-based: based on analysing a task, such as initial evaluation, prescribing, adherence counseling and so on, as a unit of work requiring specific knowledge, skills and resources, regardless of job title;

• staffing norms based on work load indicators for staffing levels.

Although most of the available staffing data are job-based (e.g. numbers of physicians, nurses), recent evaluations in the area of HIV/AIDS have taken a task-based approach. Task-based approaches provide HRH estimates that can subsequently be used to explore the effect of reassigning tasks to other cadres. WHO's Department of Human Resources for Health has proposed an approach that combines service targets (based in turn on estimates of health needs) with task analysis [14].

In this paper, data are presented in a variety of formats depending on the definitions, assumptions and calculations made by the treatment site or scale-up plan. It is important to remember that many sites were still in the process of scaling up when data were obtained and the large-scale plans have not been fully implemented. Thus the impact of these actual or proposed staffing patterns on clinical outcomes and efficiency of care remains unclear.

### Sources of HRH data

• Survey: The Millennium Project Task Force on HIV/AIDS developed a survey of existing treatment sites, the Antiretroviral Treatment Site and Affiliated Programmes Survey (ATSAP), which was then administered in collaboration with WHO. This survey covered organizational characteristics (sector, HIV/AIDS experience, collaborating agencies, scope of services), current and target activities and capacity for ART, and staffing for HIV/AIDS care and for ART-related activities. The survey also covered programme monitoring and evaluation, supply chain logistics, and major obstacles to achieving current goals or expanding. These surveys were administered in person at conferences or during WHO site visits, or filled out by the sites and returned.

• WHO site visits: WHO's Department of Human Resources for Health, in collaboration with WHO regional and country offices for Africa, South-East Asia, and the Western Pacific, performed a number of in-depth site visits to assess HRH in programmes involved in ART.

• National and programme scale-up plans and proposals

• Published literature and reports, abstracts and presentations.

• Interviews with key informants involved in scale-up activities.

From these diverse sources we extracted estimates of the number of patients an individual staff member or team would care for (patient:staff ratios). To facilitate comparison, these data were converted whenever possible into estimates of the number of staff of various kinds required to care for 1000 patients on ART. For some cadres, the potential requirements to meet the goal of having 3 million patients on ART by the end of 2005 ("3 by 5") are also discussed.

## Results

### Potential factors affecting human resource for health requirements

Many factors will affect the HRH needs, including delivery models of care (scope of services, protocols chosen, task assignment, visit frequency), site type and sector, current site resources and ART experience, provider characteristics and patient characteristics, as well as local and national support for the implementation of HIV/ART programmes. A number of these areas are discussed below.

### Delivery models of care and setting

Multiple models of care will be required to deliver care and ART at the range of settings. The current institutional settings of HIV/AIDS treatment are diverse, including public and private sectors and a wide range of levels and sites within the health care system Both the delivery model and the setting will influence HRH requirements.

#### Spectrum of care

The spectrum of care offered by sites and programmes includes: ART services only, HIV/AIDS primary care including ART management; and HIV/AIDS care integrated into a general primary care clinic [15,16]. Staffing needs will depend on the spectrum of care, on the services that are provided by the site, the services that are provided because of a referral, and the services provided by other on-site programmes (VCT, TB, etc.).

ART-only sites serve only patients eligible for or already on ART, and may or may not provide the full spectrum of HIV/AIDS primary care. Therefore, clinic staff may not be involved in providing other HIV/AIDS-related or general health services that are provided by sites adopting less specialized models. This approach maximizes the use of specialized providers trained in ART care and support, resulting in higher ART patient-to-staff ratios. These sites can also play an important role in the management of complicated ART patients from other sites, including patients with treatment failure.

In the HIV/AIDS primary care model, comprehensive care, including access to ART, is provided to all PLWHA. This model allows providers to develop some expertise in ART, but requires them to spend time caring for PLWHA who are not on ART and in the management of HIV/AIDS conditions and comorbid conditions. The level of expertise required will depend on the complexity of ART management that the site provided. In any case the provision of comprehensive care for all PLWHA will require more staff per patient on ART than a model that provides only ART. However this model allows for a more integrated approach and would facilitate access to ART and appropriate support for patients as their HIV/AIDS disease progresses.

Integration of HIV/AIDS care into a general medical clinic will further change estimates of staff requirements, since clinicians will have to care for HIV-negative patients as well as PLWHA. This model would further reduce the efficiency of ART delivery but would allow treatment to be provided at levels of the health system accessible to the greatest share of the population. Moreover, integration of ART into general primary care services, especially in high-prevalence areas, may decrease the risk that the expansion of well-funded treatment programmes will weaken other health services by competing with them for scarce skilled staff.

There is considerable variation in the models currently in use. In the WHO site visits and ATSAP surveys, eight of nine sites in Asia were designated as specialized HIV primary care sites, while in Africa nine of 19 (47%) were in HIV primary care clinics, eight were integrated into general primary care clinics, and one was in an ART-only site.

Médecins sans Frontières (MSF) has developed a classification scheme to describe a system-wide approach to expanding access to ART that is based on three levels of care. The first level provides prophylaxis of opportunistic infections (OIs), follow-up tuberculosis (TB) treatment, and simple ART initiation and OI management. The second level would include TB diagnosis, CD4 counts, more ART care and paediatric care, while the highest level would also include management of TB resistance and ART failure [15].

Staffing needs at these different levels of care would differ. As the patient's health condition becomes more complex, the staff will need more training and time to spend on the patient. Therefore increasing number of staff would be required to manage the same number of patients as clinic level and hence complexity of care increase. This model of a system of care would require that human resource needs for comprehensive ART care within an area would need to include the estimates from each level of clinic rather than just multiplying by a the number of clinics by a single estimate.

#### Staffing models and task assignment

Deciding how to designate tasks to the different types of providers and deciding what level of training will be required for each task will have some of the greatest effects on minimum and ideal staffing. These decisions are thus crucial and should be made in light of local and national shortages of specific HRH cadres. A wide range of task assignments were found from the survey and site visits (Table 1, Table 2) and in reviews of existing and proposed models.

**Table 1:** Range of cadres assigned crucial tasks at a selection of ART sites responding to the ATSAP survey

**Task**	**Sites**							
Eligibility	MD	N,C	MD	MD	C	MD	MD,CO	MD
Prescribe/initiate ART	MD	MD,MO	MD	MD	MD	MD	MD,CO	MD
Education	MD,N,Ph	N,C,CHW	N,C	MD	MD,N,C	MD,N,E,C		N,C
Adherence	MD,N,Ph	MD,N,MO,C, Ph, CHW	N,C	MD	MD,N,C	N,C,E	N,P	N,C
Follow-up (routine)	MD	MD,MO	MD, N,C	MD	MD,N,C	MD	CO,N	MD,N
Management complication/failure	MD,N,Ph		MD	MD	MD	MD	MD,CO	MD
Counselling	MD,N, chaplain	All				N,E,C		N,C

MD: physician, MO: medical officer, CO: clinical officer, N: nurse, C: counsellor, E: educator, Ph: pharmacist or pharmacy technician, NC = nurse counsellor, P = PLWH, CHW: community health worker

**Table 2:** Range of task assignments to physicians (MDs) and other providers in existing programmes providing ART visited by WHO as part of HRH assessments, or who responded to ATSAP survey

	**Asia**	**Africa**
Assess eligibility	50% MD only 38% MD and nurse	63% MD only 12% MD and other provider type 25% Other provider types only
Initiate	89% MD only	75% MD only 25% MD and other provider types
Assess toxicity/failure	22% MD only 78% MD and other provider types	All include MD, most MD only
Adherence support	54% MD or MD and other providers types 44% other provider types only.	33% MD or MD and other provider types 67% other provider types only
Routine follow-up	Not noted	88% MD or MD and other provider types 12% other provider types only

Decisions on optimizing task assignment can be facilitated by shifting from job-based staffing calculations (how many physicians) to task-based projections (how many prescribing or evaluating clinicians, who could include physicians, clinical officers (CO), medical officers (MO) or nurses). For example, a study of HRH capacity in one country in Africa estimated the MD/population ratio at 2.5/100000 [7]. But if clinical officers could perform some of the tasks traditionally reserved for physicians, the ratio of clinicians available for those tasks to population would increase to 25/100 000.

Another example can be found in a recent national scaling-up, which proposed a treatment team of one prescribing clinician (physician or MO), five evaluating clinicians (assistant MO or CO), two treatment counsellors (nurse/counsellor), three monthly counsellors (nurse/counsellor) and support personnel (pharmacist, phlebotomist, health aide, receptionist, lab technician) (*United Republic of Tanzania HIV/AIDS Care and Treatment Plan*, October 2003). For sites where a physician/MO was not available, an alternative team was proposed. This team comprised one assistant medical officer as prescribing clinician, two COs as evaluating clinicians, three nurses/counsellors, one health aide and other technical support as available and needed.

A number of programmes have already begun reassigning tasks as a result of shortages in specific classes of personnel, including assigning nurses to evaluate patients for ART and prescribe in uncomplicated cases, and shifting counselling and education from nurses to lay counsellors and trained peers (Table 1, Table 2). It is important to note that transfers of responsibilities need to be accompanied with adequate training and with supervision that reflects the standards of care for the programme. Some countries have begun a process to develop a certification process for some of these tasks to help ensure that the quality of treatment is maintained. The need for higher-level personnel such as physicians for both routine supervision and consultation for complications is crucial and should be included in HRH estimates of staffing models.

Another significant shift of task assignment has been facilitated by the growing role of lay counsellors. In many established programmes, nurses have traditionally provided the bulk of psychosocial counselling, patient education and adherence support. However, a growing number of programmes have begun using or plan to use lay counsellors trained in education and adherence promotion, as well as other skills, to provide these time-intensive services. At most stages of HIV/AIDS disease, counseling takes more time than direct clinical activities, so reassigning these duties to non-clinical staff can significantly stretch potentially limited nursing resources. For example, in one country proposal, treatment and lifestyle counsellors are predicted to spend an average of 630 minutes in year 1 for an individual on ART with CD4 <200, and 620 minutes in year 2, while prescribing and evaluating clinicians spend 90 and 150 minutes, respectively, in year 1 and only 40 minutes each in year 2 (*United Republic of Tanzania HIV/AIDS Care and Treatment Plan*, October 2003). Thus the number of patients started on ART could be increased dramatically by shifting the responsibility of nurses from counselling to evaluating patients and prescribing, although more counsellors would be needed. In many settings, however, the staff for supervision of such lay staff is not taken into consideration when HRH estimates are made.

Current programmes also vary in how other services such as adherence support and education are provided, which results in dramatically different HRH estimates. Programmes requiring directly observed therapy (DOT) generally use lay counsellors, community health workers, or trained PLWHAs. Other types of adherence support have generally been provided by nurses, but are increasing being done by trained lay counsellors, pharmacists and peer advocates. The formal involvement of PLWHAs in the ART effort is increasing and has been advocated by a number of national and international programmes, although accounting in HRH staffing numbers varies significantly [12]. PLWHAs have already assumed a variety of roles at many of the treatment sites assisted by Columbia University's MTCT-Plus programme, and at five of eight sites visited by WHO in Africa (Table 1).

#### Visit frequency

The frequency and intensity of patient visits required by ART programmes will greatly influence HRH demands. Most sites schedule more visits during the first two months on ART, with frequency generally decreasing to between monthly and quarterly. A programme that requires 12 visits in year 1 will require significantly more staff than one that requires only seven visits. The frequency and types of visits vary considerably in existing delivery models. Factors that may influence the follow-up protocol and the actual number of visits include the severity of a patient's illness, the maturity of the programme, use of DOT, national guidelines and intensity of management of initial adherence and side-effects.

In the sites visited by the WHO team as well as other programmes, the number of visits, both on- and off-site, required for ART patients in year 1 ranged from 7 to14 for programmes without DOT and 40 to 80 for those using DOTS. The minimum number of visits required to achieve maximum adherence in the short and long term is not yet known.

#### Other site/organizational characteristics

Other facility and system characteristics will influence the number and range of staff required at the site and national levels. These factors include physical space, geographical location and type of facility, sector (public, NGO, private or commercial), HIV/AIDS prevalence, health systems and programmes currently in place, degree of programme integration and linkage, government regulation and workplace conditions and efficiency. Although their impact is largely unknown, these factors should be considered when designing programmes and evaluating staffing requirements.

##### Site type and sector

The ability of programmes to adjust staffing depends on the institutional context in which they function. Typically, the public sector is subject to extensive rules and regulations governing the process by which positions are created, posted and compensated. These restrictions are often based on national staffing norms and/or standards for specific types of facilities and presumed population catchment areas. Nongovernmental organizations (NGOs), private providers and workplace programmes have fewer restrictions on staffing and task assignment, so may be better able to adapt and use staff more efficiently, as well as create new cadres to maximize HRH use. The lessons learnt from the experience of smaller, more flexible NGO-based pilot programmes on efficient use of scarce health skilled staff will be crucial as national scale-up begins.

The workplace can be an efficient place to offer care, and some workplace-based health care programmes have reported varying success in integration of HIV/AIDS care and ART into existing services [17,18]. These programmes may be able to achieve better HRH efficiency by reaching a population already on-site, although barriers, including stigma, have limited the success of some efforts. The patient-provider ratios achieved by these programmes may be most applicable to other sites that have a high concentration of patients able to come routinely for care. However, that the expansion of NGO and workplace ART programmes could further deplete public sector staff has also been raised as an additional challenge to meeting HRH needs for treatment on a national scale.

Other characteristics such as setting (rural versus urban), linkages to other programmes for supportive and other services, as well as physical space available, can also affect HRH needs (Table 3).

**Table 3:** Potential effect of selected site characteristics on human resources for health (HRH) needs

**Site characteristics**	**Potential effect on HRH needs**	
	Increase HRH	Decrease HRH

Inadequate physical space	Decreased efficiency of staff due to ineffective patient flow.	
Higher HIV/AIDS prevalence	Higher HIV/AIDS in staff and families resulting in higher absence and loss.*	Limited staff time required to ensure treating maximum number of patients.
ART integrated into general medical services	Increased staff need to be trained if significant time spent on non-HIV/AIDS/ART care.	• May improve coordination of care, particularly if multiple services co-located and make HRH use more efficient. • May be more beneficial as disease management changes focus to chronic disease management.
Rural site	Increased need for longer -distance outreach, mobile teams. Higher probability of need to integrate into other non-HIV/AIDS clinics due to smaller absolute numbers.	
Urban site	If population more transient, may require more HRH for outreach and adherence.	• Potentially easier transportation for immediate catchment area to come to clinic. • Potentially easier travel for outreach. • If higher prevalence, population may reside closer to ART site.
Weak linkages with other services (e.g. counselling, social support)	Increased HRH to provide full spectrum of care and services on-site.	

*This effect is decreasing as programmes expand ART to their staff

##### Current site resources

Current staffing levels and the scope of services already available at the care site are also important in estimating HRH needs. At sites that are already understaffed, addition of an ART programme will result in even greater demands on staff hired for the ART programme. Similarly, if few or no other essential services (TB, counselling, etc.) exist, the programme will need to plan for additional HRH to fill existing gaps. For example, in a hospital with no physician, a physician hired for an ART programme will need to spend time on inpatient and other duties.

The state of information systems and intensity of data collection and evaluation will influence HRH needs. One country's scaling-up proposal estimated that up to 50% of total staff time may be required for data management in the absence of efficient and responsive information systems. This may be particularly true in pilot or research programmes, which require more data to assess the efficacy of programme design. Finally, inadequate physical space will decrease efficiency by slowing patient flow and making it more difficult to make best use of available providers.

##### Site HIV/AIDS and ART experience

Staff and site experience in HIV/AIDS care and ART and in implementing and expanding new programmes will affect staff efficiency and efficacy. Experiences of existing programmes suggest that the number of patients on ART at new sites will likely increase slowly as staff and site gain experience and most patients are initiating ART (requiring more time than simpler follow-up). After time, a site will gain experience and a growing percentage of patients will be seen for follow-up (which requires on average less time per patient than enrolment), so the total number of patients in care will grow more rapidly for a period of time even without additional staff.

These assumptions have been incorporated into a number of national plans in estimated HRH needs (see, for example, Table 4). Two- to six-fold increases in monthly enrolment were reported in the ATSAP surveys as programmes increased their experience. Some programmes have noted that the increase in efficiency takes longer than expected, in part due to more advanced disease and other needs of presenting patients. However, as the site nears full capacity and a growing number of patients experience treatment failure or toxicities, sites' ability to continue to manage existing patients and enrol new patients will decrease unless additional resources are added.

**Table 4:** Staffing ratios over time based on proposed country strategic plan for patients on ART (staff duties include other HIV-related activities)*

	**Number of patients per staff position as planned**				
	Year 1	Year 2	Year 3	Year 4	Year 5

Prescribing clinician	534	880	1416	1947	2820
Evaluating clinician	122	189	294	389	545
Evaluating and prescribing clinicians	99	155	244	324	457
Treatment counsellor	308	405	525	660	838
Lifestyle counsellor	100	151	218	258	398
Pharmacist	629	892	1250	1634	2169
Phlebotomist	511	758	1080	1445	1950
Lab technician	517	758	11 223	1445	1950
**Total staff to treat 1000 on ART**	**27.8**	**18.5**	**12.5**	**9.7**	**7.1**

*Adapted from *United Republic of Tanzania HIV/AIDS Care and Treatment Plan*, October 2003

This effect was incorporated into projections of staffing needs in the Tanzania national plan, in which staff:patient ratios were expected to decrease over the five years from a total of 27.8 staff to treat 1000 patients to 7.1 (*United Republic of Tanzania HIV/AIDS Care and Treatment Plan*, October 2003). Similar increases in efficiency were projected across all disciplines and tasks. For example, the number of patients handled by a prescribing clinician increased from 534 patients in year 1 to over 2000 by year 5. It will be important to follow closely whether these gains are actually realized in practice as the plan is implemented.

#### Provider characteristics

Provider characteristics will also affect human resource requirements for ART. Staff training, experience, productivity, morale and attitude towards caring for PLWHAs will influence their efficiency and effectiveness (Table 5). In addition, the direct or indirect impact of HIV infection on health care workers and their families will also affect their productivity.

**Table 5:** Potential effect of selected staff characteristics on efficiency and implications for additional programme management resource needs

**Factor**	**Effect on efficiency**	**Implications**
Decreased morale	Decrease	Increase staff support and supervision, plan for retention, access to ART
Increased experience	Increase	Plan to increase retention to keep experienced staff
Increased training	Increase long term (short-term training time may decrease efficiency)	Plan for quality training on-hire (practical and didactic), continuing education, work to integrate into preclinical education in health services
Staff flexibility	Potentially increase	Provide education and support to encourage staff willing to expand/change roles to increase efficiency of the overall programme

Assumptions on efficiency (time required per visit) and productivity varied significantly among proposals and plans. This is an area in which data from large programmes are clearly needed to guide future programme estimates. In one national plan, physicians and physician assistants are expected to see 20 patients/day with 220 working days/year (*The National Strategic Plan to combat STI/HIV/AIDS*, Government of Mozambique, July 2003). Another proposal assumes 200 working days and different hours for different levels of staff (four hours a day for physicians and eight hours daily for other providers), although paperwork, training and other duties may restrict actual patient-contact hours for these other providers (*United Republic of Tanzania HIV/AIDS Care and Treatment Plan*, October 2003).

Because of high levels of HIV infection among health care workers in many countries, access to ART for staff and their families will be important for sustaining morale and decreasing staff loss from AIDS. Health care worker access to ART was designated a priority in the WHO 3 by 5 strategy. In addition, some programmes have reported significant drops in HIV/AIDS stigma after treatment for health care workers was initiated.

#### Patient characteristics

Patient characteristics will also affect HRH requirements. At start-up, a number of programmes have reported that the stage of HIV/AIDS disease and the prevalence of co-morbidities have been among the most important factors in determining the intensity of HRH demands. Most sites have focused first on the patients most in need, with high rates of advanced AIDS and complications. These individuals required more frequent and longer visits and took longer to stabilize clinically than initially expected.

Estimates of the frequency of co-morbid conditions and the need for more extensive care due to toxicities can be extrapolated from the MSF experience at a number of sites. These estimates showed that 15% of patients were on concomitant TB treatment and an additional 7% required treatment for TB after initiating ART.

ART management was further complicated by higher rates of toxicities in more advanced patients (16% had stopped at least one ARV) and by pregnancy, which occurred in 0.5% patients on ART [15]. All these conditions require at least level-2 services (notably capacity for TB diagnosis and for more complex ART management) in the MSF model. The proportion of patients who are more HRH-intensive due to clinical factors will depend on the population targeted, the stage of the HIV/AIDS epidemic in the community, access to both HIV testing and care (better access may lead more asymptomatic individuals to learn their status and present for care at an earlier stage), availability of ART at other sites in the area, and availability of CD4 counts to identify individuals with advanced immunosuppression before they develop complications.

The amount of staff time devoted to meeting the need of an individual patient will evolve. For example, more higher-level clinician time is needed at the start and first months of treatment in order to evaluate, prescribe medication and manage side effects, opportunistic infections and immune reconstitution syndrome. After this initial period, the higher-level clinician's total time requirements will decrease but the relative needs for chronic support, counselling and routine evaluation will be greater. These needs can be addressed by non-clinicians and by other cadres of health care providers, shifting the burden from clinical providers. This higher time requirement early in treatment, which may be as high as six-fold, has been reported by existing treatment sites and is reflected in country plans [19 *The National Strategic Plan to combat STI/HIV/AIDS*, Government of Mozambique, July 2003]. In one country plan, the time required per patient is assumed to decrease over the course of the first two years of ART care. (Table 6). As a result, a clinic caring for 100 individuals, all in their first year of ART, would require 9000 minutes of a prescribing clinician's time, while a clinic with 50% of patients already in year 2 would require only 6500 minutes. The total percentage of staff time accounted for by prescribing and evaluating clinicians also decreases from 25% to 11%.

**Table 6:** Estimated time required for specific cadres of providers for one patient on ART*

	**Time year 1 (minutes)**	**Time year 2 (minutes)**
Prescribing clinician	90	40
Evaluating clinician	150	40
Treatment counsellor	150	240
Lifestyle counsellor	480	360
Pharmacist	35	20
Lab technician	65	40
**Total**	**970**	**740**

*From *United Republic of Tanzania HIV/AIDS Care and Treatment Plan*, October 2003

One other important factor influencing demands on higher-level clinicians after the initial treatment period is the number of patients whose treatment regimens fail and must be changed. These individuals will require more advanced clinical management than those who remain on first-line ART. The percentage of patients experiencing treatment failure each year is currently unknown, but may range from less than 5% to more than 30% depending on prior exposure to ARVs, regimens and definitions of failure.

Other patient factors will influence the number of staff required to ensure effective ART. These include adherence rates, non-clinical support needs such as food, transportation and linkages to concrete services, counselling needs, stigma and family and community support. In addition, language gaps between patients and providers decrease efficiency and may necessitate additional staff for translation.

Finally, some populations will require more provider time during a finite period, such as pregnant women or those with tuberculosis. Children, a vulnerable group often not adequately considered in plans for ART expansion, will also require additional staff time and training.

### Estimating health workforce and patients served

Developing HRH staffing estimates will need to account for many of the factors detailed above. Other factors will include: whether HIV/AIDS patients not on ART are included in the case-load used for calculations in addition to those on ART, the methods used to determine the percentage of time spent on ART patients and the types of staff included in the totals. Available data also vary on which specific cadres (particularly lab technicians, pharmacy staff, administrative and data management staff and certain patient support personnel), were included in overall programme HRH needs. Estimates from programmes using a team-based approach will also reach different estimates than programmes that look at individual provider productivity independent of other providers. This section will explore both team and individual provider estimates at the site level.

Data from existing programmes and from proposed national plans were used to explore assumptions about HRH needs and staffing levels at existing programmes. These data are expressed either as the number of patients that an individual provider or team can treat or as the number of providers of various kinds required for 1000 patients. To begin to expand beyond the site level, estimates were explored for a two-year scale-up to treat 3 million. These estimates do not include the assumptions about changes in staff efficiency or time per patients.

#### Team-based HRH estimates

Many sites and country-level proposals take a team-based approach to staffing, incorporating multiple disciplines.

##### Existing programmes

Several different team approaches have been described (Tables 7, 8). In one example, an ART clinic in South Africa described serving 250–300 ART patients with a team of one medical officer, one nurse and 12–15 therapeutic counsellors, corresponding to six to eight medical staff and 50 counsellors for 1000 patients on ART [19]. A provincial hospital-based, high-level HIV/ART clinic used a team of one MD, 0.5 CO, one nurse, 0.5 nurse assistant, one counsellor and three PLWHAs to care for 800 individuals, implying 3.1 medical and 9.4 total staff to serve 1000. A typical team for a day at an HIV/AIDS clinic at some of the MSF sites was described as three clinicians (one MD, two nurses), two counsellors, one lab tech (variable), who can see up to 100 patients daily [15]. If one assumes that patients are seen monthly, one can infer that as few as 1.5 clinicians, one counsellor and a part-time lab technician may be able to care for 1000 patients.

**Table 7:** Selected team-based HRH needs for 1000 patients on ART from existing or proposed programme-level ART programmes

**Team**	**ART clinic (19)**	Provincial hospital**	Central hospital**
Physician or Medical Officer (MD)	3–4	1.25	2
Clinical Officer (CO)	0	0.6	-
Nurse	3–4	1.25	4
Counsellor/social worker	50	1.25	3
Other*	None noted	5	3
Total HRH per 1000 ART patients	57	9.4	12***
Total clinical staff (MD/CO/nurse) per 1000 ART patients	6–8	3.1	6

* May include administrative, nursing assistants, PLWHA not designated explicitly as counsellors

**From ATSAP survey

***also 3 community health workers, 2 pharmacist/pharmacy technicians and 3 laboratory technicians

**Table 8:** Staffing requirements for 2000 patients proposed by AIDS Healthcare Foundation**

**Service Provider**	Basic*	**Total to treat 1000 patients on ART**	**Intermediate or advanced**	**Total to treat 1000 patients on ART**
Senior physician	0	0.5 (any MD)	1	1 (any MD)
Physician	1		1	
Senior nurse	1	2 (any nurse)	1	3 (any nurse)
Junior nurse	3		5	
Front office manager	1	0.5	2	1
Administrative assistant	1	0.5	1	0.5
**Total**	**7**	**3.5**	**11**	**5.5**

*Basic care includes access to first-line ART, cotrimoxazole prophylaxis, and limited lab monitoring. Intermediate and advanced care includes second line ART as well as more advanced OI management and lab monitoring.

**H. Chang, personal communication, 2003

##### Proposed staffing plans

A number of programmes have proposed team-based approaches to delivering ART, although estimates of the number of staff required to treat 1000 patients on ART varied from 4.8 to 30.5. One hospital-based clinic in South Africa reported planning for a case-load of 1000 patients with two full-time physicians, two nurses, two counsellors, one community outreach worker, one social worker, one part-time psychologist and a team of community-based home care volunteers. Additional office staff would include a project administrator, a receptionist and a data entry clerk [20]. This model would thus require four medical staff and a total of 8.5 staff members to treat 1000 patients on ART, excluding the community-based volunteers. The AIDS Healthcare Foundation proposed a standard team of seven to serve 2000 individuals at a "basic" level (although this model offers more advanced care than similar first-line models of ART care proposed elsewhere) and a team of 11 at sites offering more complex care (Table 8) (H Chang, personal communication, 2003). These numbers do not include separate counsellors, a cadre found in many other scale-up plans, or pharmacy and lab personnel.

As noted above, in many of the country-level plans, patient-to-staff ratios change over time, reflecting increasing experience and changes in patients' clinical and ART status (starting versus follow-up). In one plan, the ratio of patients on ART to physicians is projected to increase from 88:1 in the first year to 127:1 in year 2 and finally to 180:1 by year 5. If all HIV patients in care (ART and non-ART) are included, these ratios increase to 519, 763 and 1164 patients per physician in years 1, 2 and 5, respectively (HIV/AIDS Treatment and Care Plan, Government of Rwanda, June 2003). Similar increases in patient loads are anticipated in other proposals. Table 9 summarizes projections of patient to total HRH ratios at the end of scale-up and implied numbers needed to treat 1000 on ART from four national plans.

**Table 9:** Country staffing ratios and implications for estimating staffing needs to treat patients based on country-level or other large-scale plans or proposals*

**Country**	**Annual total HRH by end of proposed scale-up**	**Target number on ART by end of proposed scale-up**	**Patient: total staff ratio at end of scale-up**	**Number to treat 1000 on ART**
Tanzania**	3014	423 050	140	7
Mozambique***^20^	1069	132 000	124	8
Rwanda^23^****	1769	57 959	33	31
Zambia^6+^	176*	24 420	139	7

*MDs, nurses, staff counsellors, lab technicians and pharmacists (if included). Administrators were not included in the country-level estimates.

**from *United Republic of Tanzania HIV/AIDS Care and Treatment Plan*, October 2003.

***from *The National Strategic Plan to combat STI/HIV/AIDS*, Government of Mozambique, July 2003

****Peer/PLWHA adherence promoters not included. Inclusion of these would decrease ratios to 4.5., *HIV/AIDS Treatment and Care Plan*, Government of Rwanda, June 2003

^+^Counselors and lab technicians not included.

Based on these ratios from proposed staffing plans, between 21 000 and 93 000 MDs, nurses and pharmacists would be required to meet the goal of providing ART to 3 million people in 2005. Considerable additional personnel will also be needed, including counsellors, lab technicians and administrative support.

A simpler team-based estimate was referenced in a recent report on an ART project in Kenya. The National AIDS and STI Control Programme estimates that 1000 patients can be treated by a team of nine to 10 health care workers, including one MD, one or two COs, one or two counselors and three to five other cadres [9]. Based on this estimate, 27 000 of these classes of personnel, including 13 500 physicians and nurses, would be required to treat 3 million patients.

#### Staffing levels for physicians, clinical officers, nurses

These estimates vary significantly because of differences in the tasks assigned to each kind of provider, in the number and types of other team members, and whether data reflected maximum capacity or the number of current patients (Tables 10, 11, 12).

**Table 10:** Patient:staff ratios based on data from selected WHO visits to active sites, 2003

**Site**	**Current no. of patients on ART**	**MDs**			**Nurses**		**Counsellors**		**Pharmacists/Pharmacy technicians**	
		N	Pts/MD	Pts/MD or CO	N	Pts/nurse	N	Pts/counsellor	N.	Pts/Ph per pharmacist/PT

Africa										
1	1100	0.2*	5500	5500	12.8	86	40	27.5	1	1100
2	1073	3	358	153	4	268	1	1073	2	537
3**	1,050	14.4	122	22	55	32	12	87	-	N/A
4	1000	3	333	333	3	333	-	N/A	-	N/A
5	833	2	417	417	5	167	-	N/A	1	833
6	704	1	704	117	4	176	2	352	1	704
7	1073	3	358	153	4	268	1	1073	2	537
8	562	1	562	562	11	52	1.28	439	1.84	305
9***	250	0.64	1953	977	-	N/A	1	250	1	250
10	105	1	105	105	10	10	10	10	1	105
11^+^	65	0.96	226	11	-	N/A	1	65	-	N/A

Asia										
1	1510	1.5	1007	1007	3.3	458	0	N/A	0.9	1678
2	935	4.3	217	217	2	468	2	468	1	935
2	800	1	800	533	1.5	533	1	800	-	N/A

*High number of nurses reported who do eligibility assessment and follow-up

*60% of time dedicated to ART-related activities

***20% of time dedicated to ART-related activities

^+^30% of time dedicated to ART-related activities

"-": not noted, N/A: not applicable

**Table 11:** Range in estimates of physicians, nurses and pharmacists to care for 1000 patients on ART from existing programmes*

**Data source**	**Physicians**		**Nurses**		**Pharmacists/pharmacy technicians**	
	Range (mean) patients/FTE	Range (mean) FTE to treat 1000 on ART	Range (mean) patients/FTE	Range (mean) FTE to treat 1000 on ART	Range (mean) patients/FTE	Range (mean) FTE to treat 1000 on ART
WHO site visits						
Africa	105–5500 (1030)	0.18–9.5 (0.97)	10–333 (141)	3–100 (7)	105–100** (588)	.91–9.5** (1.7)
Asia	217–1007 (675)	0.99–4.6 (1.48)	458–533 (486)	1.9–2.2 (2.1)	935–1678 (1306)	.59–1.1 (.77)
ATSAP survey***	250–833 (525)	1.2–4 (1.9)	25–1000 (412)	1–20 (2.4)	45–833 (329)	1.2–9.5 (3.0)
Country plans estimate^+^						
Year 1	88–534	1.9–11.4	112	8.9	558–629	1.6–1.8
Year 5	180–2820	0.35–5.6	126	7.9	623–2169	0.46–1.6

*site with low numbers of patients on ART (<150) reported highest staff to patient ratios.

**2 sites reported very high ratios of MDs to patients.

***sites reporting both MDs and medical and clinical officers had ratios of prescribing and evaluation clinicians as low as 70–286.

^+ ^Plans includes use of other cadres eligible to prescribe other medical doctors. Means could not be calculated based on data available

**Table 12:** Summary of ART experience and patient:staff ratios from ATSAP surveys^§^

							Patient-to-staff ratios***									
**Setting**	Type*	**Years ART**	**No. on ART to date**	**No. on ART**	**Target no. on ART**	**Current no. pts/mo**	**MD**	**Nurse**	**MD plus MO/CO**	**PLWH**	**Other educators**	**Counsellors**	**Ph**	**Lab tech.**	**CHW**	**Admin. or Manager**
Urban	HIV	5	1350	500	1000	30	500	250	-	333	-	333	500	500	333	0
Urban	HIV/CH	3	1000	1000	1000	90	666	1000	-	-	-	-	-	-	-	-
Urban	Med NGO+	5	800	800	1000	25	500	1000	-	-	-	-	333	500		1000
Rural	HIV DH	2	554	445	"not at target"	65	297	222	52	-	-	178	445	297	445	127
Urban	HIV CH	2	400	400	N/A	25	133	100	57	-	-	400	200	200	100	400
Urban	HIV PHC	2	288	288	N/A	N/A	192	144	-	-	-	144	-	-	-	-
Rural	Med CH	2	109	61	"could be higher"	N/A	244	-	-	-	-	-	610	610		
Rural	Med Clinic	UNK	100	100	N/A	5	300	50	-	-	-	-	-	-	-	-
Suburban	HIV/DH	4	83	40	1000	3****	400	80	-	-	80		-	-	80	
Suburban	Med DH	0.4	80	80	250	~20	250	50	-	-	-	125	500	250	250	500
Urban	HIV CH	.5	76	69	N/A	10	95	25	33	11	76	-	152	-	-	38
Urban	MTCT plus NGO+	0.5	64	64	250	5–10	833	93	139	-	250	42	833	417		104
Urban	Med CH/PHC	1	45	45	N/A	15	45	11.25	36	45	-	-	45			90
Urban	ART only/CH	1	N/A	202		35	202	67	101	-	-	Nurses	-	81		404

§When available, patient:staff ratios are based on proposed maximum site capacity

* Med: integrated into general medicine; HIV: specialized HIV only

CH: central or provincial hospital, DH: district level hospital; PHC: primary health clinic

+NGO: Nongovernmental organization-run referral tertiary care hospital

++years ART; number of years site has been providing ART

**MD: physician, MO: medical officer, CO: clinical officer, Ph: pharmacist or pharmacy technician, P=PLWH. CHW: Community health worker; N/A not available

***If target available, ratios calculated as if reached target (in bold). If the target or maximum predicted capacity was not given, ratios calculated based on current patients

**** limited by costs

##### Physicians

Although the WHO site visits in Africa found an average of 1028 patients per physician, the numbers varied enormously, from 105 to 5500 (one site reported treating 1100 patients with 0.2 MDs and 12.8 nurses). In Asia, the three largest sites (800 to 1510 patients on ART) reported patient:MD ratios ranging from 217 to 1007 (mean 675), resulting in an average of 1.5 MDs to treat 1000 on ART (Tables 10 and 11)

According to reported target capacity from the ATSAP survey, the average patient-to-physician ratio was a bit lower at 525 (range 250 to 833), implying 1.9 physicians to treat 1000. At sites that reported medical officers or clinical officers as part of the ART team, the number of patients per prescribing and evaluating clinician was as low as 70 to 286 (Table 11).

In Tanzania, where non-physicians were proposed as performing some of the ART-related evaluation activities, based on proposed ratios the number of physicians could decrease from 167 in year 1 for 16550 patients to 31 if replaced by clinical officers or nurses doing the same basic function, although additional physician time for supervision would be required (*United Republic of Tanzania HIV/AIDS Care and Treatment Plan*, October 2003). As an exercise, if we apply the assumptions from this plan to the 3 million goal, 30 000 physicians are needed if they are the only staff allowed to evaluate and prescribe. If evaluation can be done by COs or other trained and certified cadres, the numbers of physicians needed could drop to approximately 5 700. Clearly these calculations are prone to significant error, but they point to the importance of exploring the implications of reassigning tasks from the higher, more costly and more scarce cadres as programmes are designed. These choices will then have to be carefully evaluated on the basis of actual experience. But given the scarcity of medically trained workforce in most resource-poor settings, countries are likely to have little choice but to follow this model to the extent possible.

##### Nurses and clinical officers

Requirements for clinical officers and nurses at existing sites and in scale-up plans were even more variable, in part due to the wide range of assigned duties. The role for these cadres will continue to expand as shortages of physicians present a growing bottleneck to scaling up, affecting these estimates in turn. Data for clinical officers were so variable that estimates for this cadre were not attempted.

Patient-to-staff ratios for nurses from the WHO site visits ranged from as low as 10 up to 333 patients per nurse (or 3 to 100 nurses per 1000 patients on ART) even among larger programmes (Tables 10 and 11) From the ATSAP survey, ratios from sites with known target capacity again ranged from 50 to 1000 patients per nurse (average of 412), corresponding to an average of 2.4 (range 1 to 20) per 1000 patients on ART) (Tables 11 and 12). These data underline the importance of task assignment in determining the number of nurses required. In particular, as nurses' role in evaluating and monitoring patients increases, models that rely less on nurses and more on other cadres such as trained lay counsellors for counselling and adherence may be able to staff programmes more easily.

#### Staffing levels for other cadres

##### Pharmacy staff

Data were extremely limited on needs for pharmacy staff. The interpretation of the numbers was further complicated in sites where nurses are also assigned the task of dispensing medications or where it was unknown how much time pharmacists were spending on dispensing versus adherence counselling. For existing programmes that listed pharmacists or pharmacy technicians in their expansion plans, the number of patients per pharmacist/pharmacy technician ranged from 105 to 833, representing an average of 0.77 to three per 1000 patients (Table 10). In the proposed national plans, the number of patients per pharmacy staff ranged from 558 to 2169, or 0.5 to 1.8 per 1000 on ART, with lower estimates representing expected patient-to-staff ratios achieved by year 5 of scale-up. In a recent Zambia workforce study, dispensing for 10 000 patients was estimated to require 15.0 full-time equivalent (FTE) pharmacists, solely for ARVs (667 patients:pharmacist, 1.5 pharmacist per 1000 on ART), assuming monthly dispensing and an average of 8 minutes per encounter [6].

##### Treatment support and adherence support

In reviewing available data, it was often difficult to distinguish treatment and adherence support from other support activities, such as supportive counselling. This absence of more detailed task assignment information and other factors such as frequency of home visits, distance and available transportation made interpreting the range of numbers required or proposed difficult.

For treatment supporters, the number of patients per support staff range from 4 to 10, implying 100 to 250 per 1000 on ART. In an existing programme in Mozambique, one activista (a trained lay person or PLWHA who provides on-site DOT) can have a case-load up to 10 patients (100 for 1000 on ART). At Zamni Lasante in Canges, Haiti, accompagnateurs (trained lay people or PLWHAs providing home DOT and other support in rural areas) have lower case-loads (5 to 6 patients per accompagnateur, which corresponds to 167 to 200 per 1000 on ART). In one country plan, ratios were 1.6 to 5.2 patients on ART to each community/support staff (192 to 625 for each 1000 on ART) (*HIV/AIDS Treatment and Care Plan*, Government of Rwanda, June 2003). By contrast, another country plan estimated that treatment counsellors could manage 308 patients on ART in year 1, increasing to 838 patients by year 5 (3.2 decreasing to 1.2 for each 1000 patients on ART) (*United Republic of Tanzania HIV/AIDS Care and Treatment Plan*, October 2003).

Some programmes also detailed community health workers as having a variety of roles in treatment support. These programmes estimated 2.2 to 20 community health workers per 1000 on ART (50 to 445 patients per staff member). Even if programmes use community volunteers to cover some or most of these activities, some supervisory staff would still be required, although numbers required have not been established.

##### Counsellors

Counselling separate from direct treatment and adherence support was provided by a wide variety of staff. The following estimates include only staff explicitly designated as counsellors. WHO site visits found from 0.93 to 100 counselors per 1000 on ART, while the ATSAP surveys gave from 2.5 to 23.8 per 1000 on ART (average 5.5) (Tables 11 and 12). While PLWHAs were included explicitly as peer and adherence counsellors in some programmes, their role in supportive counselling was difficult to interpret, given differences in task assignments among the entire ART team. Country plans also differed, with one calling for about eight PLWHAs per 1000 patients on ART (HIV/AIDS Treatment and Care Plan, Government of Rwanda, June 2003), while another set this ratio (for "lifestyle" counsellors) at 10 per 1000 initially, decreasing to 3.2 by year 5. Clearly, deciding whether this task is assigned to nurses instead of lay counsellors will dramatically affect the number of nurses required.

##### Laboratory technicians

Inclusion of and needs for laboratory personnel varied widely, depending on which tasks were included in HRH estimates and on which lab tests were required. Estimates ranged from 80 to 1100 patients per laboratory technician (1.6 to 12.5 technicians per 1000 on ART), with most programmes that reported on laboratory technicians having one per site regardless of the number of patients.

##### Programme management

Estimates and reporting of site-level programme management also varied tremendously, based in part on different assumptions as to who would be responsible for administrative tasks (clinical, support or other staff) and whether these tasks were included in the HRH reports or estimates. When existing programmes reported on this cadre, they estimated between one and over five administrative staff per 1000 on ART. Some additional estimates come from the American Healthcare Foundation proposal, which estimated two managers for a basic-level site serving 2000 patients and three for intermediate and higher-level sites also serving 2000 patients on ART.

## Discussion

As the cost and complexity of ART continue to decline, the health workforce is emerging as one of the most important barriers to rapid scale-up in resource-limited settings. Not surprisingly, we found a wide range of ratios of patients to different cadres of providers. These ratios depend on the delivery model, site capacity, sector (NGO versus public), how long the site had been providing ART, how tasks were assigned to various cadres of health care workers, and multiple other factors. Most programmes reported or proposed that clinical teams (comprising some combination of physicians, clinical officers, nurses, counsellors, pharmacists and lab technicians) have 7 to 20 staff to treat 1000 patients on ART, although the composition of the teams varied considerably. The number of additional personnel required, especially in adherence support, varied even more widely. These data imply that the number of health care workers needed to treat 3 million people could be as few as 20 000, if only core clinical staff are included and the most optimistic estimates are followed, or more than 120 000, if a wider range of support personnel are considered. These estimates include only site-level personnel, not managers or administrative staff.

Task assignments greatly affected the number of health care workers of specific types required for a programme. Thus decisions on provider roles have the potential to significantly affect the number of patients who can be treated when there are shortages of critical cadres (e.g. physicians, nurses and pharmacists). Flexibility in task assignments to accommodate local and national human resource deficits is essential. Some progress has already been made in widening the tasks performed by nurses, increasing the role of lay counsellors, and involving PLWHA in specific aspects of prevention and care.

The data available for estimating human resource needs for ART programmes, drawn from existing treatment sites and large-scale treatment plans, are subject to several significant limitations. First, while the data from existing sites have the advantage that they derive from real experience in delivering ART in resource-poor settings, most of these sites were relatively new and considerable increases in both patient numbers and staff efficiency may be possible. The national proposals represent educated estimates as to the efficiencies that could be achieved with scaling up, but these assumptions have not yet been tested by experience. Second, the effectiveness, efficiency and sustainability of the delivery models used by existing sites have not been widely evaluated. Third, the sources used different techniques and definitions to describe current staffing ratios or predict HRH needs. In many cases, the actual percentage of time spent on ART and related activities, as opposed to other HIV/AIDS and non-HIV/AIDS-related care, was difficult to determine. As a result, many patient-to-staff ratios may underestimate the actual ART patient load per full-time-equivalent position dedicated to ART. Fourth, this paper has focused on staffing needs at treatment sites themselves and has not addressed district and central management, training and supervision, logistical support and other programme-level requirements. Finally, most of the available data were from sub-Saharan Africa, so the applicability of these estimates to other areas heavily affected by HIV/AIDS is unknown.

## Conclusion

A review of available data from existing and planned HIV/AIDS treatment programmes found substantial variation in staffing patterns and overall provider-patient ratios. While a number of factors were identified that explain some of this variation, the relative impact of each of these variables and even how to quantify them remains to be determined. Moreover, the ability to compare staffing data across sites and, ultimately, to link staffing models to effectiveness and sustainability, remains severely limited by a lack of standardized assessment tools. Streamlined systems for gathering this information and linking it to measures of programme effectiveness, staff retention and site capacity should be put in place as national programmes are launched and expanded. The time required from providers for various tasks in various settings should be monitored to inform the development of clear standards of staff productivity. These data can then help national programmes to deploy staff more efficiently and project future human resource requirements more realistically. Fortunately, the knowledge base to inform HRH planning is evolving rapidly and considerable efforts are under way to define and evaluate models of staffing. These efforts will be crucial to achieving the goal of scaling up effective HIV/AIDS treatment to reach the millions who urgently need it.

## List of abbreviations

ART: antiretroviral therapy

ARV: antiretroviral

ATSAP: Antiretroviral Treatment Site Affiliated Programmes

CO; Clinical Officer

DOT: directly observed therapy

FTE: full-time equivalent

HCW: Health care worker

HIV: Human immunodeficiency virus

HRH: Human resources for health

MO: Medical officer

MSF: Médecins sans Frontières

MTCT: mother-to-child transmission

NGO: Nongovernmental organization

OI: opportunistic illness

PLWHAs: People living with HIV and AIDS

TB: tuberculosis

WHO: World Health Organization

## Competing interests

The author(s) declare that they have no competing interests.

## Authors' contributions

LH conceived the paper, worked on survey design, did data analysis and led the writing of the paper. AF, ND and LO all actively participated in data analysis, manuscript writing and review. AF was also involved in conceiving of the project and ND participated in primary data collection. PW participated in conceiving the paper, survey design and in rewriting and editing the paper. All authors read and approved the final manuscript.
